# Norfloxacin versus alternative antibiotics for prophylaxis of spontaneous bacteria peritonitis in cirrhosis: a systematic review and meta-analysis

**DOI:** 10.1186/s12879-023-08557-6

**Published:** 2023-08-28

**Authors:** Shuailing Song, Yi Yang, Chong Geng, Zeya Tang, Chunhui Wang, Xiao Li

**Affiliations:** 1 Department of Gastroenterology, West China Hospital of Sichuan University, NO.37 GuoXue Street, Chengdu, 610041 Sichuan China; 2 Department of Laboratory Medicine, Sichuan Provincial People’s Hospital, University of Electronic Science and Technology of China, Chengdu, Sichuan China; 3 Laboratory of Gastroenterology and Hepatology, West China Hospital, Sichuan University, Chengdu, Sichuan China; 4 Department of Outpatient, West China Hospital of Sichuan University, Chengdu, Sichuan China

**Keywords:** Spontaneous bacterial peritonitis, Norfloxacin, Rifaximin, Primary prophylaxis, Second prophylaxis

## Abstract

**Background:**

Spontaneous bacterial peritonitis (SBP) is a life-threatening complication in patients with advanced cirrhosis. Prophylactic Norfloxacin used to be considered effective in SBP prevention, but in recent years its efficacy has been partially compromised by increasing quinolone-resistant bacteria. However, whether the effects of alternative prophylactic regimens are superior to norfloxacin remains controversial. The goal of this study is to compare the effects of norfloxacin with other antibiotics in SBP prophylaxis for cirrhotic patients.

**Methods:**

We systematically searched Pubmed, Embase, and Cochrane Library Databases. Two reviewers independently identified relevant random control trials (RCTs) comparing the role of norfloxacin and other antibiotics in SBP prevention.

**Results:**

Eight studies comprising 1043 cirrhotic patients were included in this study. Norfloxacin and alternative antibiotics displayed comparable effects in SBP prophylaxis, survival benefit, overall infection prevention, and safety. Subgroup analyses revealed that rifaximin prophylaxis could reduce the recurrence of SBP with fewer adverse events but failed to improve overall survival compared with norfloxacin.

**Conclusions:**

Other antibiotics are a reasonable alternative to norfloxacin in the prophylaxis of SBP. Rifaximin prophylaxis could be an alternative choose of antibiotic for SBP prevention because of its better protective effect and safety.

**Supplementary Information:**

The online version contains supplementary material available at 10.1186/s12879-023-08557-6.

## Introduction

Spontaneous bacterial peritonitis (SBP) is a deleterious and lethal complication for patients with cirrhosis and ascites [1, 2]. The one-year mortality of cirrhotic patients with SBP or a prior SBP history ranged from 30 to 50% in the natural course [2–4]. Thus, prophylaxis of nosocomial- and community-acquired SBP is pivotal for cirrhotic patients.

Patients with active gastrointestinal bleeding (GIB) and low ascitic protein concentrations were considered susceptible to SBP [5], and thus, are recommended for timely primary prophylaxis. In addition, secondary prophylaxis has been taken into consideration for patients who have experienced an episode of SBP since the one-year recurrence rate is as high as 70% in the absence of adequate prophylaxis [6]. Currently, antimicrobial prophylaxis has been suggested to prevent SBP in cirrhotic patients [3, 7]. Norfloxacin is the most widely applicated antibiotic in SBP prophylaxis [8]. A series of studies have revealed the prophylactic role of norfloxacin in primary and secondary SBP [9, 10]. However, the efficacy of norfloxacin is decreasing with the change in the pattern of causative organisms. A rising prevalence of gram-positive, quinolone-resistant, and multi-drug-resistant (MDR) bacterial are detected over the last few years [11, 12]. Possible reasons for the aforementioned shifts in the bacteriology of SBP are complex, among which extensive and long-term applications of prophylactic quinolines are unignorable components. Prolonged norfloxacin prophylaxis has even been regarded as an independent predictor of multi-resistant bacteria infections [13]. Taking bacterial resistance into consideration, antibiotic prophylaxis must be used judiciously and sparingly in patients with high risks of developing SBP, and antibiotic alternatives to norfloxacin have been explored in SBP prophylaxis. However, although several studies have suggested alternative antibiotics should be advised in SBP prophylaxis [14, 15], whether these strategies are reasonable alternatives to norfloxacin is still in debate.

Therefore, we performed the present meta-analysis primarily to compare the effects of norfloxacin and other antibiotics in SBP prophylaxis for patients with high risks of developing SBP. The secondary objectives were evaluating the survival rate, incidence of infections, and adverse events with norfloxacin and other treatment strategies.

## Methods

### Literature search

We searched papers in English language. We systematically searched clinical studies from Pubmed (1966 to March 2023), Embase (1974 to March 2023), and Cochrane Central Register of Controlled Trials (February 2023). We also checked the proceedings of annual meetings of EASL and AASLD meetings from 2018 to 2022. Studies were limited to comparing the effects of prophylactic norfloxacin and other antibiotics in the prevention of SBP. A literature search was completed by two independent reviewers (SL.S and Y.Y) using the following terms: spontaneous bacterial peritonitis, SBP, cirrhosis, ascites, infection, norfloxacin, norfloxacine, noroxin. In addition, references in relevant studies were further manually screened.

### Eligibility criteria

The following inclusion criteria were applied to screen eligible studies: (1) study was designed as a clinical randomized controlled trial (RCT); (2) enrolled cirrhotic patients were at high risk of developing SBP; (3) study assessed the effect of prophylactic norfloxacin and other antibiotic strategies in SBP prevention. A high risk of developing SBP was defined as a presence of at least one of the following factors: (i) a history of SBP; (ii) ascitic protein concentration of < 1.5 g/dL; (iii) serum bilirubin of > 43 µmol/L (2.5 mg/dL) [1, 16]. The following exclusion criteria were applied: (1) patients with malignant ascites or without advanced cirrhosis; (2) patients with active GIB; (3) patients had previously undergone liver transplantation; (4) patients received antibiotic therapy within 2 weeks of enrollment; (5) placebo and no treatment in the control group; (6) different co-interventions between the intervention arms. Two reviewers (SL.S and Y.Y) independently identified the eligible studies based on the aforementioned inclusion and exclusion criteria. Reviewers resolved discrepancies by reviewing together or consulting a third reviewer (X.L) to reach a consensus.

### Data extraction

Data of interest, including publication year, study type, population, patient age, gender, treatment drugs, and dosage, were extracted in each study by two independent reviewers (SL.S and C.G). The primary outcomes were the incidence of SBP, and the secondary outcomes were mortality, overall infection rate, and incidence of adverse events.

### Quality assessment

The methodological quality of the included studies was evaluated by two independent reviewers (X.L and CH.W) according to the Cochrane Handbook for Systematic Reviews of Interventions. The criteria included: (1) random sequence generation; (2) allocation concealment; (3) blinding of participants and personnel; (4) blinding of outcome assessment; (5) incomplete outcome data; (6) selective reporting. Each criterion was identified as having a low, high, or unclear risk. A discussion was implemented to reach a consensus in the event of a discrepancy.

### Statistical analysis

Statistical analysis was performed using Revman 5.2 software (Cochrane Collaboration, Oxford, United States). All results were presented as pooled risk ratios (RRs) and 95% confidence intervals (CIs). Potential bias was checked by the funnel plot method with Egger’s test. To heighten the robustness of the results, the pooled RRs and 95% CI were all calculated by the random effects model. Heterogeneity was evaluated by χ^2^ tests with *p* values and *I*^*2*^ statistic values. We reported heterogeneity when the *p* value was less than 0.1 and further explored potential heterogeneity. Subgroup analysis was also employed based on the study design.

## Results

### Study characteristics and quality assessment

**Fig. 1:**
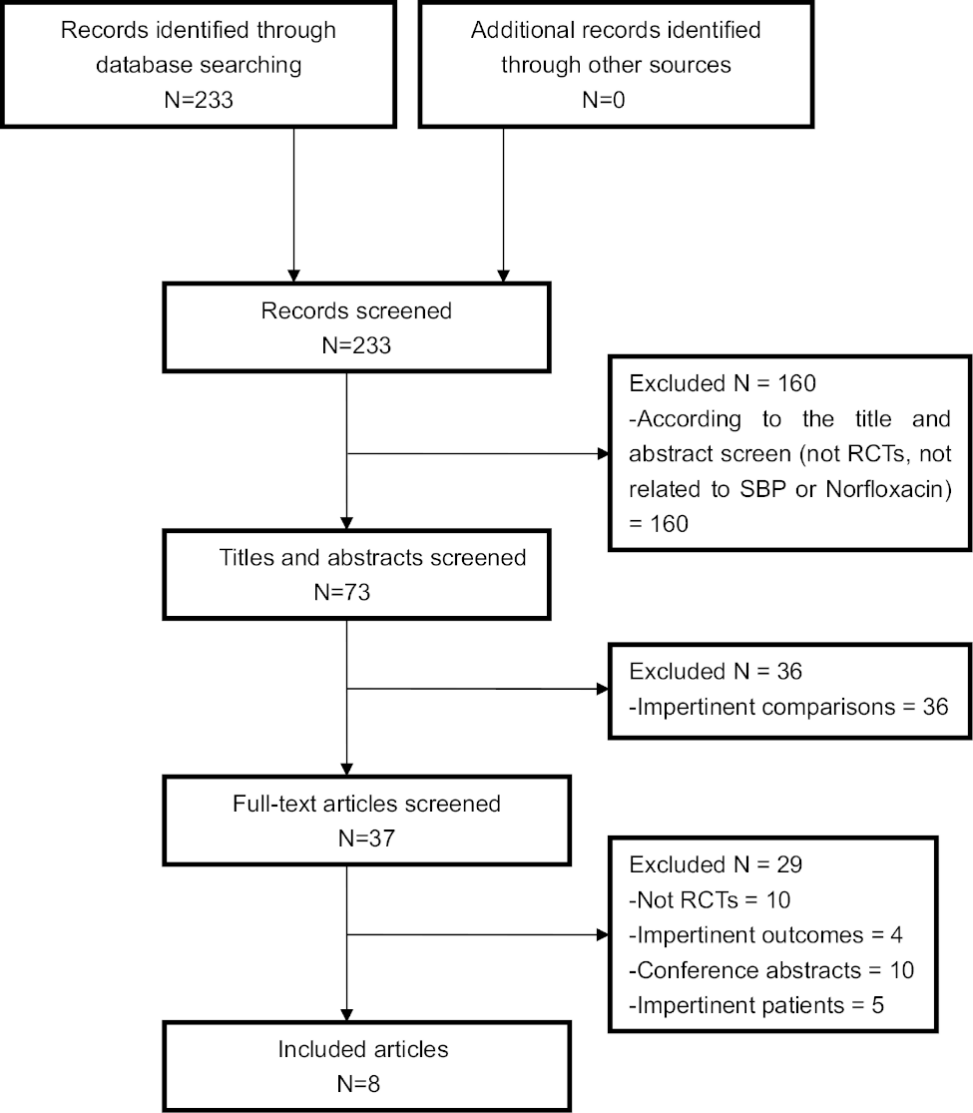
Flowchart diagram for study selection in meta-analysis. “impertinent” means not meeting the inclusion criteria

The details of the screen flow are summarized in Fig. 1. And a PRISMA checklist was provided in Additional file 1: Table S1. Eight RCTs [1, 14, 15, 17–21] comparing the prophylactic effects of norfloxacin and alternative antibiotics in the prevention of SBP were included. Of those, four RCTs compared norfloxacin with rifaximin with respect to the prevention of SBP [15, 17, 19, 20], two RCTs showed different efficacy between norfloxacin prophylaxis and trimethoprim-sulfamethoxazole (T-S) prophylaxis [1, 14], and the other two studies reported the incidence of SBP after taking prophylactic norfloxacin in comparison with rufloxacin [18] and ciprofloxacin [21], respectively. Additionally, four studies evaluated the effects of antibiotics for both primary and secondary prophylaxis [1, 14, 20, 21], with one for primary prophylaxis [17] and two for second prophylaxis [15, 19]. The characteristics of the included studies are summarized in Table 1. A detailed quality assessment of the included studies is described in Additional file 2: Figure S1. In addition, the funnel plots for SBP, mortality, incidence of overall infection, and incidence of adverse events were shown in Additional file 3: Figure S2, and Egger’s test indicated that there was no significant publication bias.

**Table 1:** Characteristics of the included randomized controlled trials (RCTs)

Study	Center	Type of prophylaxis	Antibiotics for prophylaxis	Main cause of cirrhosis (percentage) ^†^	Number of patients (total/male) ^†^	Mean age (year) ^†^	Mean ascitic total protein (mg/dl) ^†^	Mean TB (mg/dl) ^†^	Mean creatinine (mg/dl) ^†^	Maximum follow-up period
Bauer, 2002	Multiple	Secondary	Norfloxacin	HBV or HCV (67%)	40/26	59	NA	2.90	1.10	12 months
			Rufloxacin	HBV or HCV (62%)	39/29	62	NA	3.00	1.00	
Alvarez, 2005	Multiple	Both	Norfloxacin	Alcohol (28%)	32/20	52	960	4.94	1.76	547 days
			T-S	Alcohol (44%)	25/18	44	1370	3.53	1.00	
Lontos, 2014	Multiple	Both	Norfloxacin	Alcohol (47.5%)	40/32	53	NA	NA	NA	12 months
			T-S	Alcohol (37.5%)	40/28	54	NA	NA	NA	
Mostafa, 2015	Single	Secondary	Norfloxacin	HCV (100%)	30/16	57	NA	2.36	1.68	6 months
			Rifaximin	HCV (100%)	40/20	56	NA	2.46	1.70	
Assem, 2016	Multiple	Primary	Norfloxacin	HCV (93.6%)	78/56	58	930	2.80	1.60	6 months
			Rifaximin	HCV (90.2%)	82/60	55	890	2.80	1.50	
			Combination	HCV (94.9%)	79/60	57	930	3.00	1.60	
Elfert, 2016	Single	Secondary	Norfloxacin	NA	131/68	54	1100	2.76	1.24	6 months
			Rifaximin	NA	131/74	54	1000	2.69	1.27	
Yim, 2018	Multiple	Both	Norfloxacin	HBV (50.0%)	62/46	56	1030	3.35	0.95	12 months
			Ciprofloxacin	Alcohol (48.4%)	62/44	55	1050	3.94	0.88	
Praharaj, 2022	Single	Both	Norfloxacin	NA	62/48	46	1050	3.55	1.05	6 months
			Rifaximin	NA	54/41	48	1250	3.65	1.10	

^†^ The upper column represents the norfloxacin group, and the lower column represents the other antibiotic group

T-S: trimethoprim-sulfamethoxazole; HBV: hepatic B virus; HCV: hepatic C virus; NA: not applicable; TB: total bilirubin

### Overall incidence of SBP

**Fig. 2:**
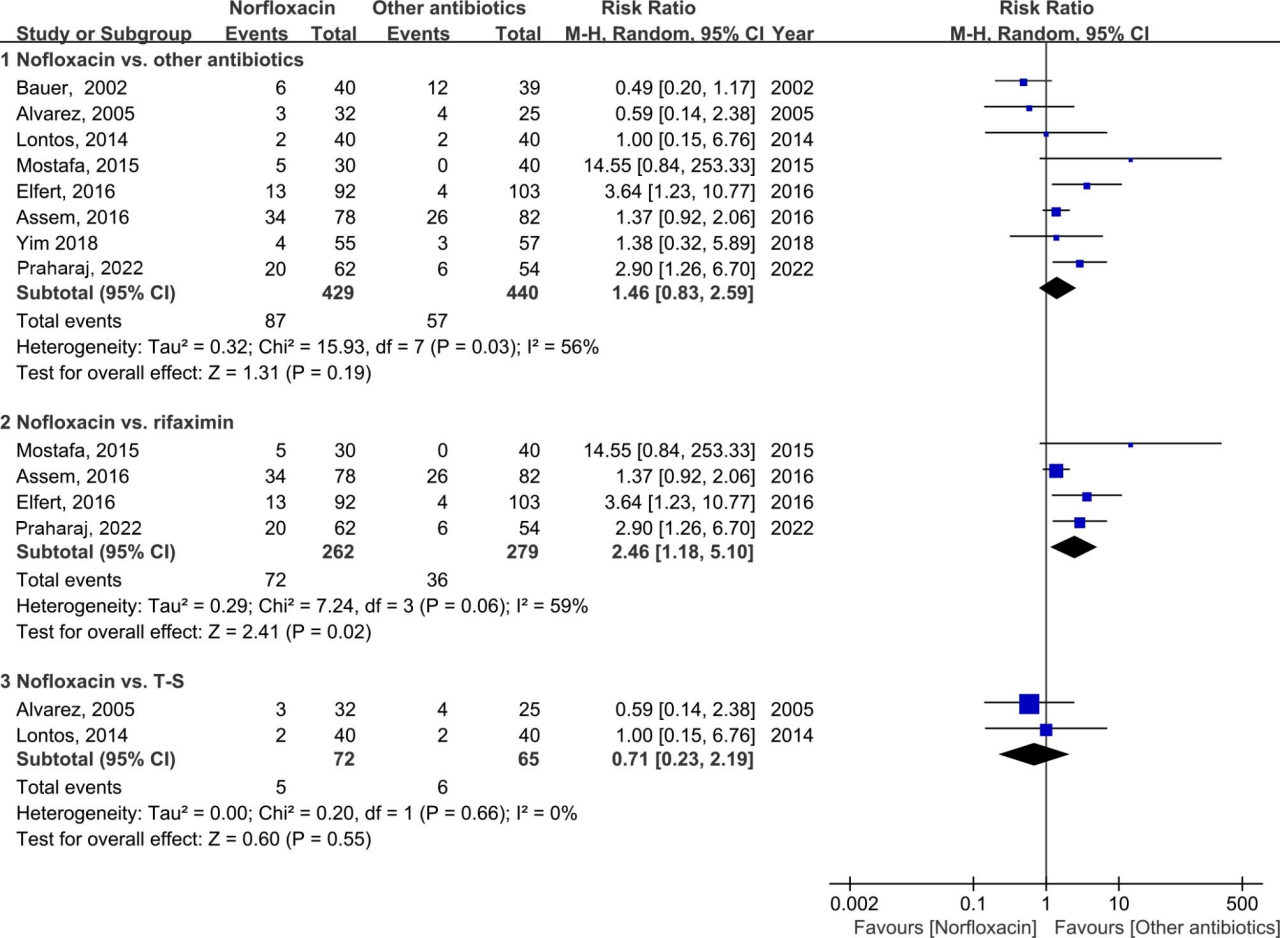
Forest plot of the overall incidence of SBP between the norfloxacin group and the other antibiotic group. SBP, spontaneous bacterial peritonitis

First, we compared the effects of norfloxacin with alternative antibiotics in the prevention of overall SBP. As shown in Fig. 2, the overall incidence of SBP was comparable between prophylactic norfloxacin and alternative antibiotics (RR: 1.46; 95% CI: 0.83, 2.59; *p* = 0.19). In subgroup analyses, the data showed that the effect of rifaximin prophylaxis was much superior to norfloxacin in SBP prevention (RR: 2.46; 95% CI: 1.18, 5.10; *p* = 0.02). In addition, pooled analyses from two trials [1, 14] indicated that T-S prophylaxis could not reduce the incidence of SBP when compared with norfloxacin (RR: 0.71; 95% CI: 0.23, 2.19; *p* = 0.55).

### Primary and secondary prophylaxis of SBP

**Fig. 3:**
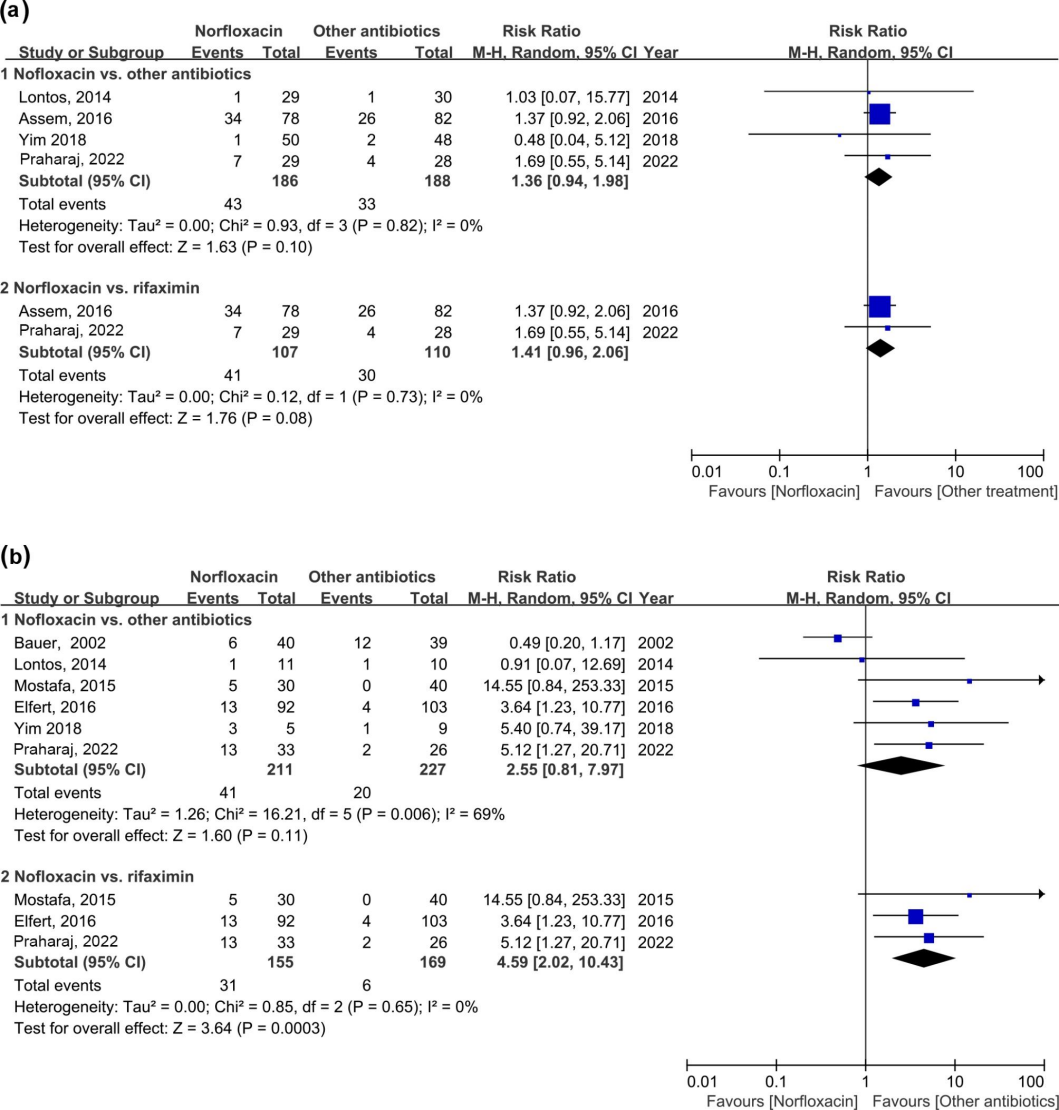
Forest plot of the incidence of SBP between the norfloxacin group and the other antibiotic group in the primary prophylaxis (a) and the secondary prophylaxis (b). SBP, spontaneous bacterial peritonitis

As the risk of SBP occurrence is different in cirrhotic patients who had an episode of SBP or not, we further evaluated the effects of norfloxacin and other antibiotics in primary and secondary SBP prophylaxis. Four studies [1, 17, 20, 21] compared norfloxacin to other antibiotics for primary SBP prophylaxis and five studies [1, 15, 18–20] for secondary prophylaxis. The results showed the effects of other antibiotics were comparable to norfloxacin for primary SBP prophylaxis (RR: 1.36; 95% CI: 0.94, 1.98; *p* = 0.10) and secondary SBP prophylaxis (RR: 2.55; 95% CI: 0.81, 7.97; *p* = 0.11) (Fig. 3a-b). Subgroup analyses indicated that for primary prophylaxis, there was a decreased tendency in SBP occurrence with rifaximin treatment compared to norfloxacin (RR: 1.41; 95% CI: 0.96, 2.06; *p* = 0.08). In addition, prophylactic rifaximin significantly decreased the reoccurrence of SBP in the secondary prophylaxis, compared with norfloxacin (RR: 4.59; 95% CI: 2.02, 10.43; *p* = 0.0003) (Fig. 3b).

### Mortality

**Fig. 4:**
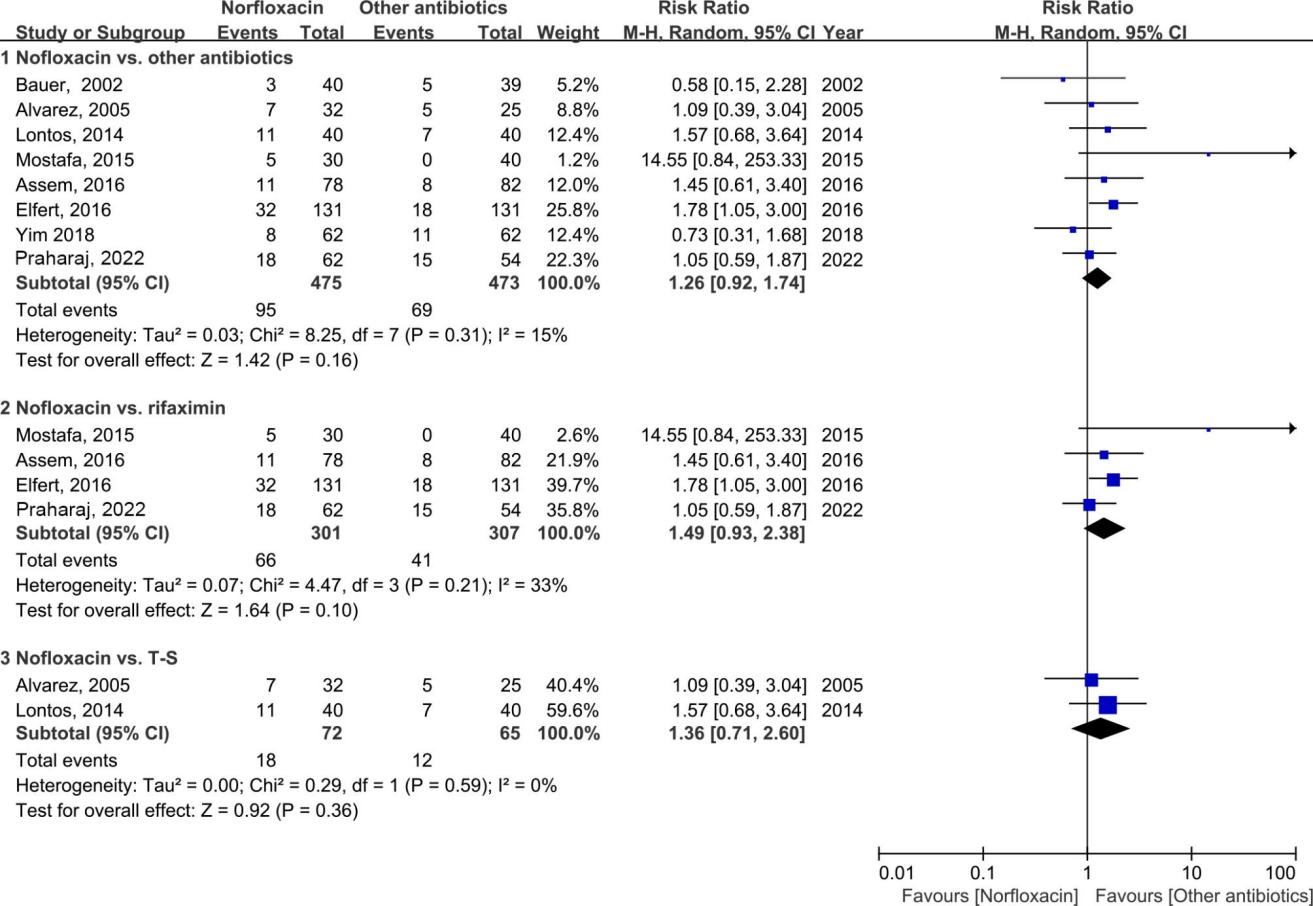
Forest plot of the morbidity

Eight studies [1, 14, 15, 17–21] evaluated the mortality regarding norfloxacin and other antibiotics in the prophylaxis of SBP (Fig. 4). Overall pooled analyses indicated patients with other antibiotic prophylaxis achieved comparable survival benefits compared with norfloxacin (RR: 1.26; 95% CI: 0.92, 1.74; *p* = 0.16). Specifically, subgroup analyses showed that rifaximin prophylaxis failed to decrease mortality compared to norfloxacin (RR: 1.49; 95% CI: 0.93, 2.38; *p* = 0.10). A consistent result was found when comparing T-S with norfloxacin prophylaxis (RR: 1.36; 95% CI: 0.71, 2.60; *p* = 0.36).

### Incidence of overall infection

**Fig. 5:**
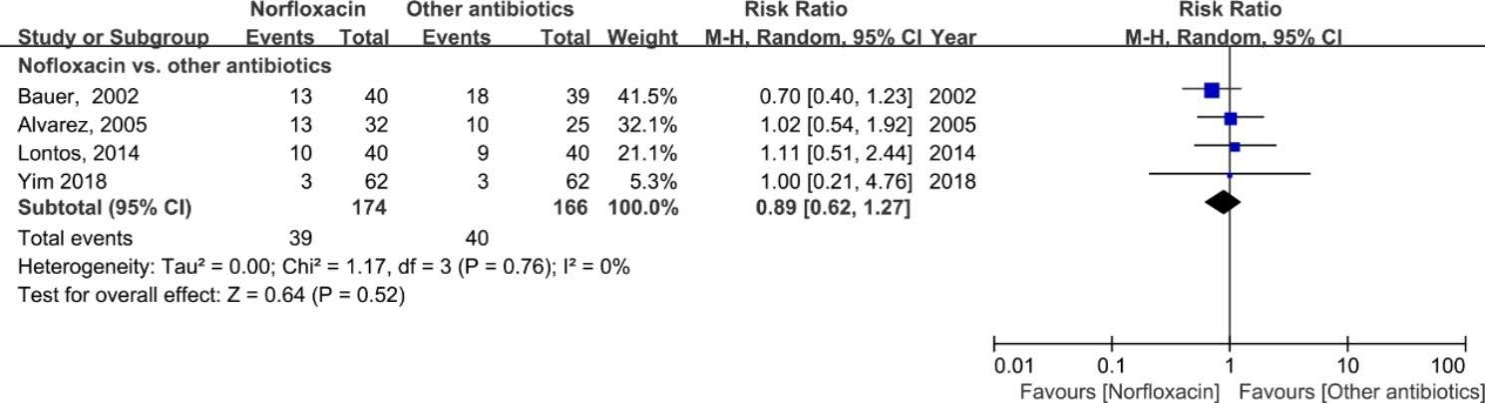
Forest plot of the overall infection

Of note, four studies [1, 14, 18, 21] reported the incidence of overall infections (Fig. 5). The incidence of overall infections in prophylactic norfloxacin was similar as other antibiotics in SBP prevention (RR: 0.89; 95% CI: 0.62, 1.27; *p* = 0.52).

### Adverse events

**Fig. 6:**
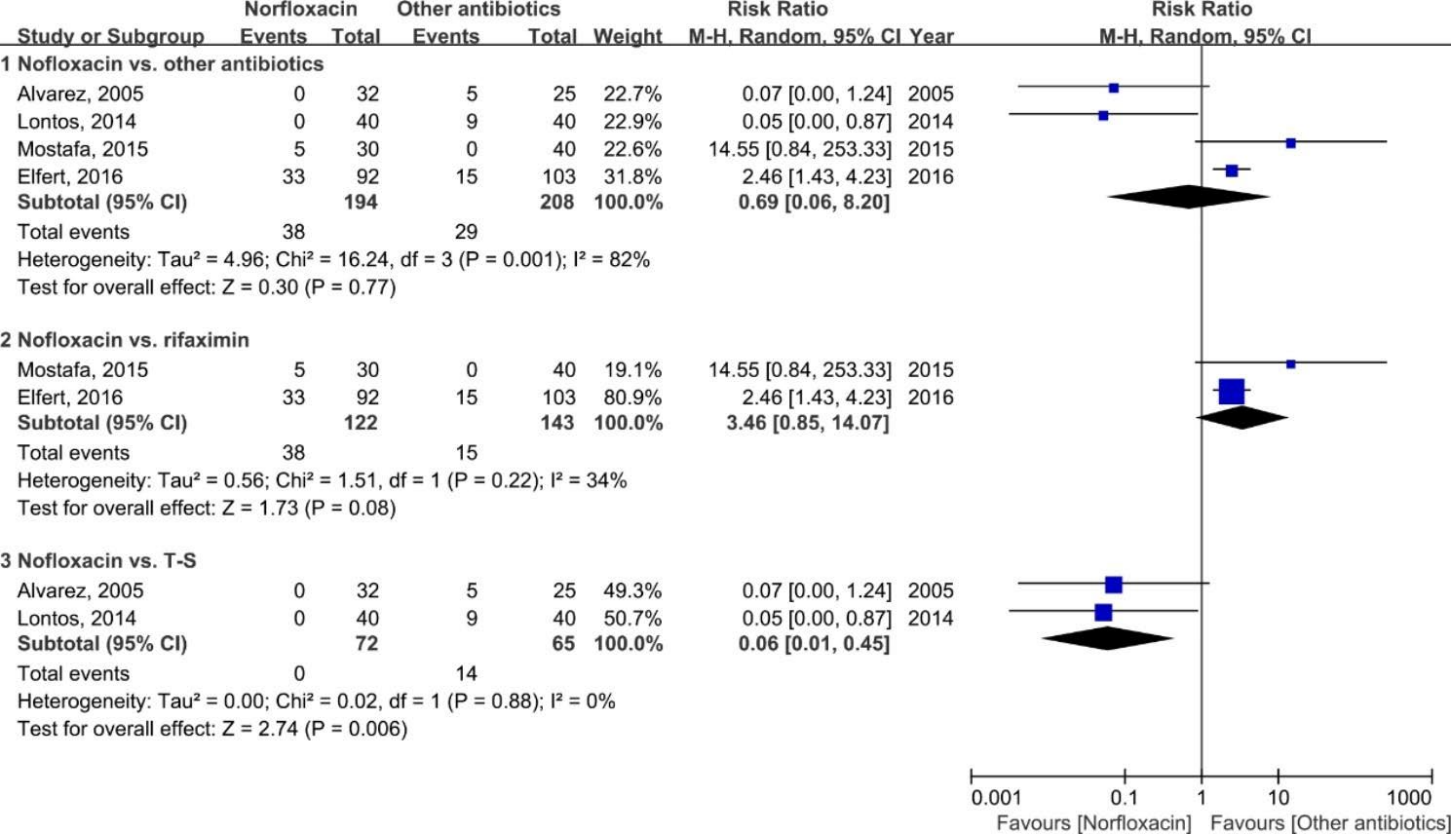
Forest plot of the adverse events

To comprehend the safety of prophylactic antibiotics in SBP prevention, we evaluated the incidence of adverse events reported in studies. As shown in Fig. 6, the incidence of adverse events in patients with norfloxacin prophylaxis revealed no significant difference compared with other antibiotics (RR: 0.69; 95% CI: 0.06, 8.20; *p* = 0.77). The Chi-square test indicated statistical heterogeneity existed among the studies (*p* = 0.001, *I*^*2*^ = 82%), and subgroup analyses were further performed. The results demonstrated that the incidence of adverse events associated with rifaximin prophylaxis displayed a reduction tendency that was almost marginally significant (RR: 3.46; 95% CI: 0.85, 14.07; *p* = 0.08). However, patients with prophylactic T-S had an obvious increase in the incidence of adverse events compared to norfloxacin (RR: 0.06; 95% CI: 0.01, 0.45; *p* = 0.006).

## Discussion

SBP is a frequent and severe complication in cirrhotic patients with ascites. Even when appropriate treatments are adopted, acute kidney injury and acute-on-chronic liver failure occur in 54% and 35%-60% of patients, respectively [22–25]. About 30–50% of cirrhotic patients could die from SBP within one year, as mentioned before [2–4]. Mechanically, intestinal bacterial overgrowth, impaired intestinal barrier function with consequent bacterial translocation, and systematic immune dysregulation are generally considered to be involved in the pathophysiology of SBP [26]. Traditionally, gram-negative bacilli are the major pathogenic bacteria of SBP, with *E. coli* and *Klebsiella* being the most frequently isolated bacteria [27, 28]. Therefore, norfloxacin has been widely applied to prevent SBP because of its action against gram-negative bacteria and its low systematic availability. However, an alteration has occurred to the pattern of pathogens in SBP, characterized by an increase in gram-positive bacteria and drug-resistant bacteria, which is attributed to the massive use of prophylactic quinolones, the widespread use of invasive procedures, the increasing administration of broad-spectrum antibiotics, and the broadening criteria for hospitalization in intensive care units [29]. And this shift in the bacteriology of SBP has challenged the traditional antibiotic strategy represented by norfloxacin [30]. In the current study, based on publication years of studies, we compared the prophylactic effects of norfloxacin in SBP prevention before [14, 18] and after 2010 [1, 15, 17, 19–21]. It is worth noting that the incidence of SBP in patients receiving norfloxacin prophylaxis increased from 12.50 to 21.85%. This finding was consistent with the previous meta-analysis that pointed out that the incidence rate ratios (IRRs) for placebo versus norfloxacin significantly decreased from 15.35 to 1992 to 2.13 in 2015 [31]. It implies that the positive treatment effect of norfloxacin decreased over time. Given the dismal prognosis of SBP and the altered epidemiology of bacterial infections in cirrhosis, it is essential to adjust prophylactic strategies. Alternative antibiotics had been proposed to prevent SBP in specific cirrhotic patients. However, the prophylactic effects of alternative strategies relative to norfloxacin are still ambiguous.

In the current meta-analysis, we enlisted eight RCTs [1, 14, 15, 17–21] to compare the preventive effects of norfloxacin to those of other antibiotics, including rifaximin, T-S, rufloxacin, and ciprofloxacin. Norfloxacin and other antibiotics had comparable overall occurrences of SBP. However, rifaximin-treated patients had better prophylactic effects for SBP prevention than those using norfloxacin (12.90% vs. 27.48%, *p* = 0.02), according to subgroup analysis results. In addition, we further analyzed the effects of antibiotics in primary and secondary SBP prophylaxis. Subgroup analysis of the available data indicated that the prophylactic effects of norfloxacin were comparable to those of other antibiotics. Of note, an overt decreased tendency without significance was observed in rifaximin intervention for primary SBP prophylaxis compared with norfloxacin (27.27% vs. 38.32%, *p =* 0.08). Interestingly, for secondary SBP prevention, rifaximin exhibited more robust prophylactic effects than norfloxacin (3.56% vs. 20%, *p =* 0.0003). These findings suggested that rifaximin was a promising and effective alternative to norfloxacin in SBP primary and secondary prevention. Rifaximin, as a gut-selective, low microbe-resistant antibiotic with a broad anti-bacteria spectrum, had been proposed as an oral alternative antibiotic to norfloxacin to prevent SBP [15, 20]. Mechanism studies showed that rifaximin exerts a limited impact on microbial composition in cirrhosis [32–34]. In contrast, norfloxacin has been proven to be more effective than rifaximin in avoiding episodes of bacterial translocation, at least in experimental cirrhosis [32]. Maybe the very subtle changes in the microbiome composition induced by rifaximin are sufficient to improve the metabolism of the host in cirrhosis. In addition, our meta-analysis showed that rifaximin exploited its advantage over norfloxacin mainly for SBP secondary prophylaxis. This may be because, compared with norfloxacin, rifaximin exerts a more significant impact on the microbial environment of secondary infections, where different isolated bacteria from first infections, increasing fungal infections, and multi-drug resistant bacteria are usually found [35]. More underlying mechanisms need to be investigated. Similarly, it could explain why a comparable efficacy was detected between T-S prophylaxis and norfloxacin prophylaxis in the present study, as the majority of quinolone-resistant strains are also resistant to T-S [36]. From our analysis, rifaximin seems to be an attractive alternative to norfloxacin to reduce SBP recurrence.

The overall prognosis of cirrhotic patients with high risks of developing SBP is poor. In the present study, the pooled analyses indicated consistent mortality with norfloxacin and other antibiotic prophylaxis. Consistently, subgroup analysis indicated that rifaximin exerted a comparable impact to norfloxacin on the survival benefit of cirrhotic patients, despite its advantage over norfloxacin in SBP secondary prophylaxis. Of note, other liver-related complications like acute kidney injury [37, 38], acute-on-chronic liver failure [39], and nosocomial infection [40] are also related to poor outcomes in liver cirrhosis. These components should be taken into consideration when we talk about the survival benefit brought by antibiotic prophylaxis for SBP.

Patients with cirrhosis and ascites were susceptible to developing systemic infections. The mortality rate in cirrhotic patients with infections obviously increased [41]. Usually, cirrhotic patients accompanied by infections would have a dismal prognosis, and gut microbiota alternation and translocation were considered to be associated with systematic inflammation and an undesirable prognosis [42, 43]. Hence, intestinal decontamination drugs were suggested to prevent SBP in cirrhotic patients. Previous meta-analyses have reported the positive effects of norfloxacin in reducing overall infections in cirrhotic patients when compared with placebo or no-treatment groups [44]. However, in the present study, we reported non-superior effects of alternative antibiotics to norfloxacin in the prevention of overall infections in cirrhotic patients. Of note, given the limited studies included, this result necessitated further verification by more RCTs.

Drug safety as well could not be ignored in the application of antibiotics. Here, four studies [1, 14, 15, 19] compared and reported the adverse events of norfloxacin with rifaximin or T-S, respectively. Side effects such as headache, dizziness, nausea, abdominal pain, and flatulence were occasionally observed in cases using norfloxacin or rifaximin; and anorexia, rash, and vomiting were individually reported in cases using T-S. Almost all of the adverse events were mild and disappeared after drug withdrawal or expectant treatment. Overall, our results illuminated comparable incidences of adverse events with other antibiotics and norfloxacin. What should be particularly pointed out was that the adverse events in T-S prophylactic patients obviously increased compared to norfloxacin (21.54% vs. 0%, *p =* 0.006), suggesting more drug safety should be considered when prophylactic T-S is attempted in SBP patients. Of note, additional subgroup analysis revealed patients with rifaximin prophylaxis were likely to experience fewer adverse events compared with those receiving norfloxacin (10.49% vs. 31.15%, *p =* 0.08). We speculated that the high safety of rifaximin might be due to its minimal intestine-absorbed property.

We evaluated the risk bias of all the included RCTs to assess the quality of evidence. Notably, the evaluation of risk bias was somewhat different from other articles. For instance, the risk of bias for blinding of outcome assessment in Alvarez’s RCT was considered high in our study, unclear in Komolafe’s article [45], and low in Soni’s article [46]. We suspected this inconsistency may be caused by the low reliability of the risk of bias tool [47]. Therefore, improved guidelines for the RoB tool and revisions to the tool are needed.

Our study has certain limitations. Because only a few RCTs have compared rifaximin versus norfloxacin, the strength of the positive results presented in our meta-analysis is undermined by methodological drawbacks. Additionally, the results are affected by heterogeneous and low-quality studies. Therefore, more well-conducted and larger RCTs are needed.

## Conclusions

In summary, the present meta-analysis updated and comprehensively demonstrated the effects of norfloxacin vs. other prophylactic antibiotics in SBP prevention. Generally, for cirrhotic patients with high risk, rifaximin prophylaxis for SBP showed greater efficacy and safety. Thus, we suggested that the use of rifaximin or a combination with norfloxacin might have more advantages in high-risk patients for the prophylaxis of SBP compared with norfloxacin alone. This updated meta-analysis could contribute to developing appropriate antibiotic strategies and provide evidence to support the use of rifaximin in the prevention of SBP.

### Electronic supplementary material

Below is the link to the electronic supplementary material.

Supplementary Material 1

Supplementary Material 2

Supplementary Material 3

## Data Availability

The datasets used and/or analysed during the current study are available from the corresponding author on reasonable request.

## Abbreviations

CIs: Confidence intervals
GIB: Gastrointestinal bleeding
IRRs: Incidence rate ratios
MDR: Multi-drug-resistant
RCTs: Random control trials
RRs: Risk ratios
SBP: Spontaneous bacterial peritonitis
T-S: Trimethoprim-sulfamethoxazole
