# Supplementary material for: Norfloxacin versus alternative antibiotics for prophylaxis of spontaneous bacteria peritonitis in cirrhosis: a systematic review and meta-analysis
Source: BMC Infect Dis. 2023 Aug 28;23:557. doi: 10.1186/s12879-023-08557-6 (PMC10463656; doi:10.1186/s12879-023-08557-6)
Supplement: Supplementary file 3 — Supplementary Material 3 [file 12879_2023_8557_MOESM3_ESM.pdf]

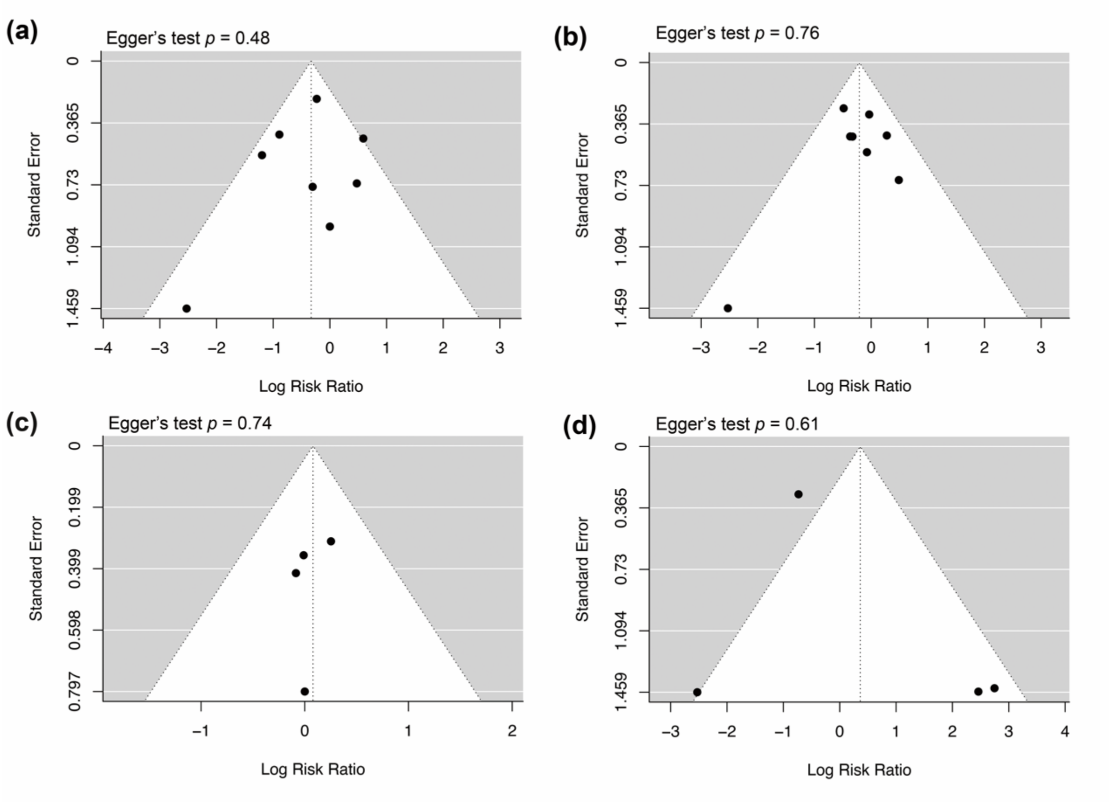

Figure S2. Funnel plots to assess publication bias for (a) SBP, (b) mortality, (c) incidence of overall infection, and (d) incidence of adverse events. SBP, spontaneous bacterial peritonitis.
